# Intracerebral administration of Interleukin-12 (IL-12) and IL-18 modifies the course of mouse scrapie

**DOI:** 10.1186/1746-6148-2-37

**Published:** 2006-12-27

**Authors:** Paolo Pasquali, Romolo Nonno, Maria Teresa Mandara, Michele Angelo Di Bari, Giovanni Ricci, Paola Petrucci, Silvia Capuccini, Claudia Cartoni, Agostino Macrì, Umberto Agrimi

**Affiliations:** 1 Department of Food Safety and Animal Health, Istituto Superiore di Sanità, viale Regina Elena 299, 00161, Rome, Italy; 2 Department of Biopathological Veterinary Science, Veterinary Medicine School, Università degli Studi di Perugia, Perugia, Italy

## Abstract

**Background:**

Prion diseases are characterised by a neurodegenerative pattern in which the function of immune system remains still elusive. In the present study, we evaluate if an exogenous treatment with Interleukin-12 (IL-12) and IL-18, able to activate microglia, is able to affect scrapie pathogenesis.

**Results:**

Cytokines injected intracranially, induced a strong inflammatory response characterised by TNF-α production and microglia activation. Two groups of mice were injected intracerebrally with high dose of ME7 strain of scrapie containing IL-12 and IL-18 or sterile saline. Cytokines-treated mice showed a more pronounced accumulation of PrP^Sc ^in brain tissues at 90 days post-inoculation and a shorter mean survival times than untreated mice.

**Conclusion:**

We can conclude that intracerebral administration of IL-12 and IL-18 can modulate scrapie pathogenesis possibly through a microglia-mediated pattern.

## Background

Prion diseases are characterised by a neurodegenerative pattern represented by neuronal loss, vacuolation of neurons and neuropil, and astrocytosis. They are characterized by the accumulation of an abnormal protein, named PrP^Sc^, which is formed postranslationally from the normal isoform (PrP^C^). To date, the agent causing TSEs is still incompletely characterized, although PrPSc is believed to be its major if not unique constituent [1] which when present as it has recent as small PrP^Sc ^aggregates are the most efficient initiators of TSE disease [2].

A classical inflammatory response associated to the infection is lacking, as evidenced by the absence of proinflammatory cytokine upregulation [3], of a polymorphonuclear leukocyte infiltration and perivascular cuffing of mononuclear cells around cerebral vessels or any humoral or cellular immune response to infection [4,5]. Nevertheless, there is a large body of evidence that immune system is involved somehow in the pathogenesis of the disease [6,7]. Indeed, strong circumstantial evidence of the involvement of immune system is provided by the fact that a significant number of genes associated with the immune response have shown increased expression in scrapie-infected mouse brains [8,9], that mice have shown reduced susceptibility to scrapie after steroid administration [10] and splenectomized mice have shown prolongation of incubation time to scrapie [11]. In analogy, in other experiments it has been reported that susceptibility to scrapie was enhanced by aspecific stimulators, such as phytohemagglutinin (PHA) [12].

In mouse models, follicular dendritic cells (FDC) are involved in scrapie replication and neuroinvasion following peripheral inoculation of scrapie agents is strongly impaired in animals lacking these cells [13-15]. More recently, it has been shown that also dendritic cells acquire PrP^Sc ^in vitro, and transport directly into lymphoid tissues in vivo, suggesting that dendritic cells can serve as a cellular bridge between the gut lumen and the lymphoid compartment [16]. From that scenario it can be envisaged a "Trojan-Horse" effect of these cells of the immune system even if little is still known about their activity in scrapie. It has been suggested that at least one mechanism by which immune system is tightly involved in prion infection is through the primary uptake of prion protein, probably representing a narrow bridge between the portal of entry of the prion protein and the peripheral and the central nervous system [17]. Nevertheless, it was also observed that skin-derived Langherans-like cells have been shown to be able to degrade PrP^Sc ^in vitro [18] and this findings contribute to the hypothesis that monocytic cells can also have a protective effect against prions. All together, these results clearly indicate that the relationship between cells and prions is far to be completely elucidated and possible dynamic relationships can determinate different outcomes. The interplay between scrapie agents and the immune cells is even less understood in central nervous system. Microglia, which are the immunocompetent cells that can be considered the equivalent of dendritic cells [19], seem to be involved in the pathogenesis of the disease [20] but their effective role is still under debate. There is a large body of evidence, however, which suggests that glial cells, through their secreted proinflammatory cytokines contribute to prion-related neurodegenerative processes [21-27]. Overall the gene array data demonstrate the presence of a strong inflammatory reaction and stress response in the late phase of the disease [28].

In the present study, we evaluate if the onset of the disease can be modified by exogenous treatment with cytokines known to act synergistically in promoting a proinflammatory response [29], in order to better clarify the role of immune cells in scrapie in the central nervous system of mice.

## Results

### Effect of intracerebral administration of IL-12 and IL-18 on central nervous system

Mice were treated with cytokines or sterile saline, according to protocols, and after 6 or 24 hours TNF-α and IFN-γ production, along with PrP^C ^expression, were monitored in brain homogenates. In parallel an immunohistochemical analysis of GFAP and F4/80 expression was performed on paraffin embedded brain tissue.

**Figure 1:**
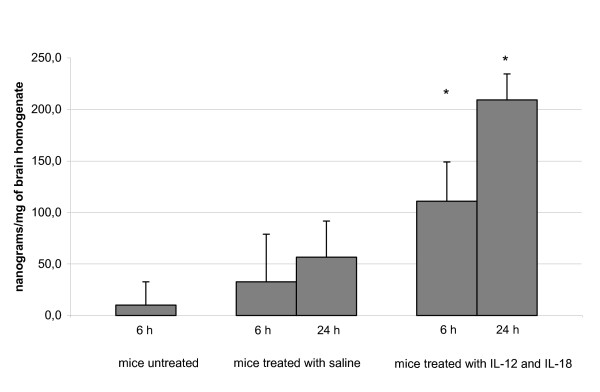
**IFN-γ production**. IFN-γ production, detected in brain homogenates, from untreated mice or mice intracerebrally inoculated with saline or IL-12 and IL-18, sacrificed at different time after treatment. * values statistically different from those obtained in mice treated with saline at the same time.

**Figure 2:**
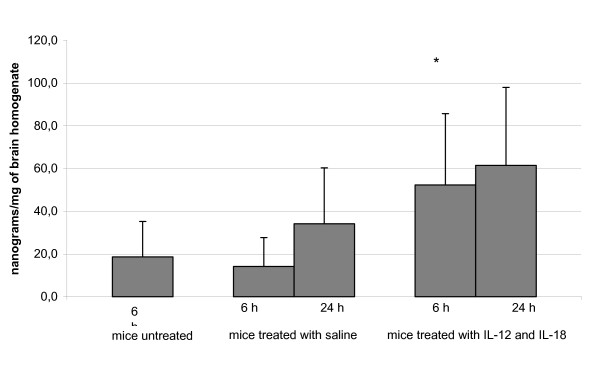
**TNF-α production**. TNF-α production detected in brain homogenates, from untreated mice or mice intracerebrally inoculated with saline or IL-12 and IL-18, sacrificed at different time after treatment. *values statistically different from those obtained in mice treated with saline at the same time

IFN-γ and TNF-α were produced in mice stimulated both with IL-12 and IL-18, even if with slight different patterns. Mice inoculated with sterile saline produced a moderate amount of IFN-γ and TNF-α but differences with untreated mice were difficult to address due to the high variability of the measured values. Mice injected with IL-12 alone did not showed a significant increase of IFN-γ, and only a slight increase of TNF-α (data not shown). Mice injected with IL-18, showed a significant increase of IFN-γ with at a lesser extent also an increase of TNF-α (data not shown). Mice inoculated with IL-12 and IL-18, finally showed a strong increase in the production of both IFN-γ and TNF-α, with a more prominent effect after 24 hours of exposure (figures 1 and 2).

The expression of PrP^C ^was assessed by western blot analysis in individual mice 6 and 24 hours after treatment and compared with that of untreated mice. The administration of IL-12 and IL-18 did not induce any change in the expression of PrP^C^, neither at 6 or 24 hours after their intracerebral administration (data not shown).

Immunohystochemistry was performed in basal nuclei, thalamus, cerebellum and brain stem of mice enrolled in the experiment. Immunostaining for GFAP and F4/80 was used as indirect assessment of activation of astrocytes and microglia, respectively.

GFAP positive astrocytes were present in the normal, untreated brains and their number increased after treatment with sterile saline. This increase was mainly evident in the brain stem. The pattern of GFAP expression did not change after treatments with IL-12 and IL-18 when compared to saline (Table 1).

F4/80 positivity was barely detectable in brain parenchyma of normal, untreated mice. Reactive monocytes located near brain vessels were also present and were considered perivascular macrophages. In mice treated with saline there was a very slight increase in the number of activated microglia, almost constant in all brain sections. Mice treated with IL-12 and IL-18, showed a marked increase in F4/80 microglia and perivascular macrophages expression throughout the brain areas observed (Table 1). Our findings indicate that IL-12 and IL-18 were able to exert a substantial activation of microglia 6 and 24 hours after their intracerebral administration.

### Effect of intracerebral administration of IL-12 and IL-18 on the course of scrapie infection

**Figure 3:**
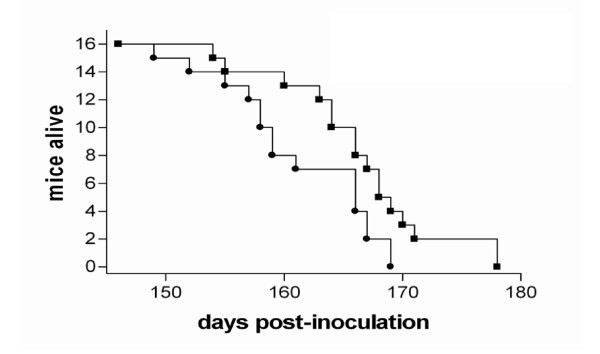
**Survival of scrapie infected mice**. Survival times after intracerebrally injection of scrapie ME7-derived brain homogenate (10% w/v in sterile saline) of mice treated with IL-12 and IL-18 (circles) or with saline (squares).

To assess if an early activation of microglia, contemporary to intracerebral scrapie inoculation, is able to modify the course of scrapie infection, two groups of 22 mice were infected with scrapie and treated with IL-12 and IL-18 or sterile saline, respectively, according to protocols. After 90 days 6 animals from each group were sacrificed to indirectly assess the progression of the infection by determining PrP^Sc ^brain accumulation. The remaining mice were monitored to assess the mortality rate (Fig. 3). All inoculated mice developed unequivocal clinical signs of scrapie, starting after 125 days post-inoculation. There was a general tendency of mice in the IL-12 and IL-18 treated group to develop clinical symptoms earlier than untreated mice. IL-12 and IL-18 treated mice started to dye at day 149 post-inoculation, while untreated mice started at day 154 post-inoculation. Throughout the observation period, there was a clear tendency of IL-12 and IL-18 treated mice to die before the untreated mice. Mean survival times (± SEM) were 161,1 ± 1,5 for IL-12 and IL-18 treated mice and 166,3 ± 1,7 for untreated mice. Survival curve analysis indicated a statistically significant difference between the two groups (two-tailed P value < 0,05).

**Figure 4:**
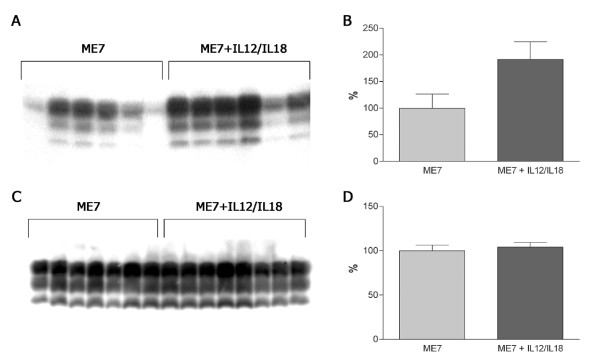
**PrPsc evaluation**. Western-blot patterns (A, C) and semiquantitative analysis (B, D) of brain homogenates of mice sacrificed at 90 days after infection (A, B) or terminally sick (C, D).

At 90 days post-inoculation PrP^Sc ^accumulation was quite variable among individual mice (Fig. 4A); mice inoculated with IL-12 and IL-18, however, showed a more pronounced accumulation of PrP^Sc ^in brain tissue than untreated mice, with a statistical analysis that closely approached the significance (two-tailed P value = 0,054, Fig. 4B). To further corroborate this finding and due to the variability observed, we pooled the brain homogenates of mice sacrificed at 90 d.p.i. and compared PrP^Sc ^in the pooled homogenates of the two groups. The pooled homogenates from the IL-12/IL-18 treated mice showed a more than doubled PrP^Sc ^content compared with that of untreated mice (data not shown).

The PrP^Sc ^brain accumulation was very invariable in terminally sick mice and similar pattern and quantity of PrP^Sc ^deposition were observed in IL-12 and IL-18 treated and untreated mice (Fig. 4C and 4D).

The results obtained using a single dose of scrapie inoculum were then re-evaluated using the same dose of cytokines and different doses of scrapie inocula (data not shown). This experiment confirmed that the exogenous treatment of IL-12 and IL-18 induced a reduction of survival time in mice inoculated with 10^-2^, 10^-3 ^and 10^-4 ^ME7 dilutions.

## Discussion

IL-12 is a heterodimeric interleukin produced by antigen presenting cells as macrophages, dendritic cells or microglia. It plays a central role in cell-mediated immune response. Recent findings show that IL-12 actively plays an important role also in central nervous system but its influence seems to be a double-edged sword having both a detrimental effect observed in experimental allergic encephalomyelitis or multiple sclerosis [30] and a protective role against neurotropic viruses [31]. IL-12 acts in a paracrine fashion in the cascade of events that lead to a Th1-like immune response but it has been seen that it may play also an autocrine effect on microglia [32,33].

IL-18 is a pleiotropic pro-inflammatory cytokine produced by antigen presenting cells. It is a newly-described cytokine that has been shown to be essential for IFN-γ production [34]. There is no direct evidence of autocrine effect of IL-18 on central nervous system. However, the finding that mouse peritoneal macrophages stimulated in vitro with recombinant murine IL-18 induced TNF-α, IL-1α and IL-1β [35], suggests that IL-18 can exert also an autocrine effect on microglia which represent the main population of macrophage-like cells in the brain. This fact is further corroborated by the results of recent studies showing that IL-18-gene-disrupted mice had impaired microglial activities [36]. Due to the well-known synergistic effect of IL-12 and IL-18 [29], we firstly tested the effect of the direct intracerebral administration of these two cytokines.

We found that intracerebral administration of IL-12 and IL-18 induces a production of TNF-α and IFN-γ in brain tissue, at 6 and 24 hours after administration. In addition, we found that IL-12 and IL-18 induced also the stimulation of macrophage-like cells.

These findings do not clarify the complete scenario of events following inoculation but support the concept that IL-12 and IL-18 are able to induce a broad spectrum of effects, comprising the activation of microglia as showed by the increased cytokine production and especially by increased immunoreactivity to F4/80 in brain tissues. It is unclear if the cellular activation is due to direct effect of IL-12 and IL-18, mimicking an autocrine pattern or it is the effect of activated T lymphocytes that, in turn, produce IFN-γ and hence stimulate microglia through a classic paracrine pattern.

We further investigated the effect of combined IL-12 and IL-18 on scrapie pathogenesis. We found that a single intracerebral cytokine administration, contemporary to the scrapie inoculum, is able to affect the course of scrapie pathogenesis. In mice inoculated with high prion dose (10% ME7-derived brain homogenate), the treatment with IL-12/IL-18 induced a small but statistically significant reduction of mean survival time. This effect was paralleled by an increased brain accumulation of PrP^Sc ^at 90 days post inoculation, but not at the terminal stage of the disease. Our findings complement those obtained by Thackray et al. [37], who found that IL-10 knock-out mice show a marked reduction of incubation period intracerebral or peripheral scrapie infection. IL-12 and IL-18 on one side and IL-10 on the other, in fact, can be considered counteracting molecules in the macrophage-like cells.

In our study we did not observe any effect on PrP^C ^expression after intracerebral administration of IL-12 and IL-18. We assessed PrP^C ^expression by measuring its content in brain homogenates, a method that mostly account for neuronal expression of PrP^C^, but can be not adequate for measuring subtle variations in other cell types of the brain. In scrapie, incubation time can be markedly reduced in transgenic mice overexpressing PrP^C ^[38] and prion protein expression has been reported to be cytokine-inducible in some human neural cell lines [39]. The prion protein is expressed at high levels in the central nervous system and this mainly depends on high expression in neurons [40], but PrP^C ^is also expressed in microglia and astrocytes [41,42]. Taken together these considerations let us to conclude that the reduction of the incubation period we observed in mice treated with IL-12 and IL-18 is not explainable by modification of PrP^C ^expression in neurons; however we can not exclude that IL-12 and IL-18-activated microglia overexpress PrP^C^, as it is true for IFN-γ-activated human CD14+ monocytes [43]. On the basis of the effect observed after intracerebral administration of IL-12 and IL-18, we can suppose that IL-12 and IL-18-activated microglia favor the progression of the disease when high prion doses were used. In this scenario it is possible to postulate two different mechanisms that can explain our findings. The first is that activated microglia have an increased capability to uptake scrapie agents and to present them to neurons, implying that microglia act as bridge to reach neurons. There is evidence, in fact, that besides lympho-reticular cells [11,44,45] also mononuclear phagocytic cells [46,47] support replication of prions and may mediate transmission of infection from periphery to central nervous system. In addition, more recent findings indicate that a localized early inflammatory response takes place within the central nervous system of mice intracerebrally inoculated with brain homogenates from normal or scrapie infected mice [48,21]. It suggests that inflammatory cells, such as microglia, can act in the early response to prion invasion after intracerebral scrapie inoculation. Finally, it has been found that purified microglia from Creutzfeldt-Jakob Disease-infected mice show infectivity comparable to that of starting brain homogenate even if express less PrP [49]. The second mechanism is that IL-12 and IL-18 administration increases the ability of microglia to damage central nervous system, which led to a more precocious neuronal dead. This hypothesis is based on the finding that widespread neuronal degeneration is likely to induce microglial activation which, in turn, may further contribute to neurotoxicity [50] by releasing inflammatory cytokines such as IL-1, IL-6 and TNF-α that contribute to the development of pathological findings [27]. It is worth noting that the reduction of the incubation period observed was obtained with a single interleukin administration at the time of scrapie inoculation. It is difficult to postulate that such a treatment would exert its effects in the late phase of the disease, i.e. 3 to 5 months later. It is our opinion, therefore, that these findings give credit to the hypothesis that IL-12 and IL-18-activated microglia are more efficient to uptake and to present scrapie agents to neurons.

Incubation time of scrapie is inversely proportional to the logarithm of the concentration of the inoculum as reported by us and others [51,52]. This pattern would be consistent with a process of exponential growth of the scrapie agent that cause disease onset at a threshold level. Our results are reminiscent of such a process because we observed a more pronounced accumulation of PrP^Sc ^at 90 days post-inoculation and a decreased incubation time in IL-12 and IL-18-treated mice compared to the control animals. On the other hand, all mice with terminal disease showed very similar levels of PrP^Sc ^brain accumulation irrespective of treatment, implying that cytokines administration did not interfere in the interplay among scrapie agent replication, PrP^Sc ^accumulation and neuronal degeneration in the late phase of the disease. These findings strongly indicate that IL-12 and IL-18 inoculated mice developed the disease before control mice because of a faster kinetic of PrP^Sc ^accumulation and a more rapid progression of the disease.

## Conclusion

we provided evidence that intracerebral administration of IL-12 and IL-18 stimulated microglia in central nervous system and that a single treatment contemporary to intracerebral scrapie injection is able to affect the course of scrapie infection. Our findings provide insight into the process involved in the central nervous system pathogenesis of scrapie and contribute to shed renewed light on the interplay between immune system and scrapie agents.

## Methods

### Animals and pathogen

The ME7 strain was kindly provided by Moira Bruce of the Neuropathogenesis Unit at Edinburgh.

C57BL/6 female mice were obtained from Charles River, Italy, and used in the experiments at nine weeks of age. They were maintained in barrier housing with filtered inflow air in a restricted-access room and in pathogen-limited conditions. They were fed a commercial diet and water was provided ad libitum. All mice were acclimatised for 1 week prior to experimentation. In the first set of experiments, mice were employed in groups consisting of 5 animals each. Mice were anaesthetised and injected stereotactically in striatum (with coordinates: Bregma 0.38 mm)(Keith B.J. Franklin and Gorge Paxinos – The Mouse Brain in Stereotaxic Coordinates. Academic Press) with 20 μl of mouse recombinant Interleukin-12 (IL-12) (1000 ng) and/or IL-18 (500 ng), purchased from R&D Systems, MN, USA. Controls consisted of injected with sterile saline, or uninoculated mice. Following treatment, mice were allowed to recover under a warm lamp. Mice were sacrificed at 6 and 24 hours after treatment and brains were rapidly removed. Brains were longitudinally cut, for each brain one hemisphere was fixed in buffered formalin and the other was snap frozen on liquid nitrogen.

### Immunological analyses

The frozen hemispheres were successively weighed and homogenised (20% W/V) in PBS containing protease inhibitors (Complete Mini, Roche). Mouse Tumor Necrosis Factor-alfa (TNF-α), and Interferon-γ (IFN-γ) were detected in duplicate in brain homogenates by enzime-linked immunosorbent assay (ELISA) according to the manufactures instructions of a commercial kit (R&D Systems, MN). For PrP^C ^detection, 50 μl of each brain homogenate was diluted to 200 μl in lysis buffer (PBS containing 0,5% Nonidet P40 and 0,5% Sodium Deoxycholate, final concentration) and incubated 20 min at room temperature. The samples were then centrifuged at 1000 g for 5 min in a microcentrifuge and supernatants were added with equal volumes of 2× NU-PAGE sample buffer (Invitrogen), heated at 95°C for 10 min, centrifuged in a microcentrifuge at 12,000 rpm for 5 min and subsequently subjected to electrophoresis and western blotting as described below.

The other hemispheres were fixed for 5 days in 10% buffered formalin. Coronal sections corresponding to basal nuclei, thalamus, cerebellum and brain stem were embedded in paraffin wax. Serial sections were performed at 5 μm and stained with Haematoxylin-Eosin (HE) or submitted to immunolabelling (IHC). For the latter, two different tests were performed in order to detect astrocyte proliferation and activation using the mAb anti-glial fibrillar acidic protein (GFAP) and to detect microglia activation using the mAb anti-F4/80.

For GFAP immunolabelling, deparaffinized sections were treated with 3% H_2_O_2 _in methanol for 10 min at room temperature to abolish endogenous peroxidase activity and, after heat induced antigen retrieval (citrate buffer 0,01 M, pH 6.0, 1 × 5 min in microwave oven, 750 watts), incubated 30 min with polyclonal rabbit anti cow GFAP [1:500, Dako, Glostrup, Denmark]. Immunoreactivity was detected with avidin-biotin-complex (ABC) method [*LSAB+*^®^, Dako Corporation, San Francisco, USA] as suggested by the suppliers.

For F4/80, after heat induced antigen retrieval treatment (citrate buffer 0,01 M, pH 6.0, 20 min, in steamer), monoclonal rat anti F4/80 antigen [1:100, Serotec Ltd, Oxford, UK] was applied for 30 min. Immunolabelling was performed with a Polymer-Immunocomplex method [*Picture*™ *Plus*, Zymed Laboratories Inc., San Francisco, USA], in accordance with the manufacturer's recommendations.

Reactions were visualized with 3, 3' diaminobenzidine [Dako Corporation, Carpinteria, USA] and Mayer's haematoxylin was used as counter stain. Tris-buffered saline (TBS, 50 mM Tris-HCl, 150 mM NaCl, pH 7.6 with 0.05% Tween 20) was used as washing and antibody diluent buffer. Slides were analysed by two independent operators who ranked the degree of reactivity in 20 microscopic fields.

In the second set of experiments two groups of 22 mice were anaesthetised and injected stereotactically in striatum (with coordinates: Bregma 0.38 mm) (Keith B.J. Franklin and Gorge Paxinos – The Mouse Brain inStereotaxic Coordinates. Academic Press) with 20 μl of ME7-derived brain homogenate (10% w/v in sterile saline) containing IL-12 (1000 ng) and IL-18 (500 ng) or sterile saline. Six mice of each group were sacrificed at 90 days post-inoculation to evaluate the presence of PrP^Sc^. The remaining 16 mice for each group were clinically scored weekly and observed daily from the first appearance of clinical signs up to terminal disease, to determine the survival times. Brains from mice sacrificed at 90 days after infection or dead due to scrapie were removed, snap frozen in liquid nitrogen and kept at -80°C until use. The frozen brains were weighed and homogenised (10% w/v) in PBS containing 2% Sarcosyl. After incubation at room temperature for 20 min, proteinase K was added to 50 μl from each sample to a final concentration of 200 μg/ml. Samples were then incubated for 1 hour at 37°C with gentle shaking followed by the addition of PMSF to a final concentration of 3 mM. Finally, equal volumes of 2× NU-PAGE sample buffer (Invitrogen) were added and the samples were heated at 95°C for 10 min, centrifuged in a microcentrifuge at 12,000 rpm for 5 min and subsequently subjected to electrophoresis and western blotting. Ten μl of samples were loaded onto 12% bis-Tris polyacrylamide gels (Invitrogen). Electrophoresis was carried out at 200 V for 40 min and Western blotting performed on polyvinylidene difluoride membranes (Millipore) at 100 mA per gel for 40 min in a semidry blotting apparatus (Trans-Blot SD, Bio-Rad). The blots were blocked in PBS containing 0.1% Tween 20 and 5% non-fat milk powder for 1 h. Both, PrP^C ^and PrP^Sc ^were detected with the monoclonal antibody SAF84 (0,2 μg/ml, provided by J. Grassi, CEA, France), for 1 h at room temperature. Horseradish peroxidase-conjugated goat anti-mouse IgG (1:5,000 for 1 h, Sigma) was used as secondary antibody. The membranes were developed with the enhanced chemiluminescence method (Supersignal, West Femto, Pierce). Chemiluminescence detection was performed with the VersaDoc imaging system (Bio-Rad) and signals were quantified with the Quantity One software (Bio-Rad).

### Statistical analysis

Differences of TNF-α and IFN-γ production was evaluated by analysis of variance with significance when P ≤ 0.05. Differences in PrP^Sc ^accumulation or PrP^C ^expression were assessed by unpaired t test by calculation of two-tailed P values. The significance of different mortality curves was assessed by survival curve analysis, calculated with the Prism software (Graphpad), which compares survival curves using the logrank test (Mantel-Haenszel test).

## Authors' contributions

PP designed study, analysed data, drafted manuscript, and planned co-ordination among contributors

RN participated in the study design and sample collection, performed biochemical analysis, participated in drafting the manuscript

MTM planned co-ordination in histological and immunohistological analyses, participated in drafting the manuscript

MADB managed experimental animals, performed clinical examinations, collected sample

GR performed histological and immunohistological analyses, participated in drafting the manuscript

PP participated in sample collection and immunological analyses

SC performed histological and immunohistological analyses

CC participated in sample collection and contributed to biochemical analysis

AM revised manuscript and provide scientific advice

UA: contributed to the study design and data analysis, participated to drafting manuscript.

All authors read and approved the final manuscript

## Figures and Tables

**Table 1:** Reactivity to GFAP and F4/80 in brains from mice inoculated with saline and IL-12/IL-18.

	**Astrocytes (GFAP)**		**Microglia (F4/80)**	
**Treatment**	6 hours	24 hours	6 hours	24 hours

Saline	4/16 (25%)	2/14 (14,3%)	1/16 (6,2%)	2/14 (14,3%)
IL-12/IL-18	2/16 (12,5%)	5/15 (33,3%)	7/15 (46,7%)	8/14 (57,1%)

Mice were sacrificed 6 or 24 hours after intracerebral inoculation. Observation was made in different areas of brain, by different independent observers. Numbers are referred to the fields where it was observed an increase of reactivity upon the examined fields.
